# Triphen­yl(tetra­hydro­furan)­aluminium(III)

**DOI:** 10.1107/S1600536808032091

**Published:** 2008-10-11

**Authors:** Chi-Ren Chen, Han-Mou Gau

**Affiliations:** a Department of Chemistry, National Chung Hsing University, Taichung 402, Taiwan

## Abstract

In the title compound, [Al(C_6_H_5_)_3_(C_4_H_8_O)], the Al atom has a distorted tetra­hedral geometry. The C—Al—C angles range from 113.25 (7) to 116.27 (8)°, much larger than the O—Al—C angles, which range from 103.39 (7) to 103.90 (6)°. The tetra­hydro­furan ring adopts an envelope conformation. The crystal packing is stabilized by C—H⋯π inter­actions.

## Related literature

For general background, see: Chen *et al.* (2007 ▶); Ku *et al.* (2007 ▶); Wu & Gau (2006 ▶). For related structures, see: Barber *et al.* (1982 ▶); De Mel & Oliver (1989 ▶); Jerius *et al.* (1986 ▶); Malone & McDonald (1967 ▶).
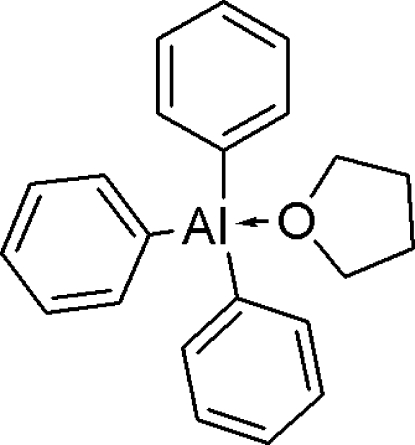


## Experimental

### 

#### Crystal data

[Al(C_6_H_5_)_3_(C_4_H_8_O)]
*M* *_r_* = 330.38
Monoclinic, 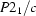

*a* = 9.649 (2) Å
*b* = 12.966 (3) Å
*c* = 16.038 (4) Å
β = 104.210 (4)°
*V* = 1945.2 (8) Å^3^
*Z* = 4
Mo *K*α radiation
μ = 0.11 mm^−1^
*T* = 293 (2) K
0.58 × 0.42 × 0.21 mm

#### Data collection

Bruker SMART 1000 CCD diffractometer
Absorption correction: multi-scan (*SADABS*; Sheldrick, 1996 ▶) *T*_min_ = 0.799, *T*_max_ = 1.000 (expected range = 0.781–0.977)
10682 measured reflections
3804 independent reflections
2971 reflections with *I* > 2σ(*I*)
*R*_int_ = 0.023

#### Refinement

*R*[*F* ^2^ > 2σ(*F* ^2^)] = 0.048
*wR*(*F* ^2^) = 0.162
*S* = 1.33
3804 reflections
217 parameters
H-atom parameters constrained
Δρ_max_ = 0.27 e Å^−3^
Δρ_min_ = −0.23 e Å^−3^

### 

Data collection: *SMART* (Siemens, 1996 ▶); cell refinement: *SAINT* (Siemens, 1996 ▶); data reduction: *SAINT*; program(s) used to solve structure: *SHELXS97* (Sheldrick, 2008 ▶); program(s) used to refine structure: *SHELXL97* (Sheldrick, 2008 ▶); molecular graphics: *SHELXTL* (Sheldrick, 2008 ▶); software used to prepare material for publication: *SHELXTL*.

## Supplementary Material

Crystal structure: contains datablocks I, global. DOI: 10.1107/S1600536808032091/ci2683sup1.cif

Structure factors: contains datablocks I. DOI: 10.1107/S1600536808032091/ci2683Isup2.hkl

Additional supplementary materials:  crystallographic information; 3D view; checkCIF report

## Figures and Tables

**Table 1:** Selected bond lengths (Å)

Al1—O1	1.8972 (13)
Al1—C1	1.9783 (18)
Al1—C13	1.9800 (18)
Al1—C7	1.9809 (19)

**Table 2:** Hydrogen-bond geometry (Å, °) *Cg*1 is the centroid of the C1–C6 ring.

*D*—H⋯*A*	*D*—H	H⋯*A*	*D*⋯*A*	*D*—H⋯*A*
C16—H16⋯*Cg*1^i^	0.93	2.78	3.654 (4)	156
C19—H19*A*⋯*Cg*1^ii^	0.97	2.81	3.600 (4)	139

Symmetry codes: (i) 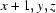; (ii) 
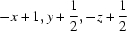
.

**Fig. 1.**
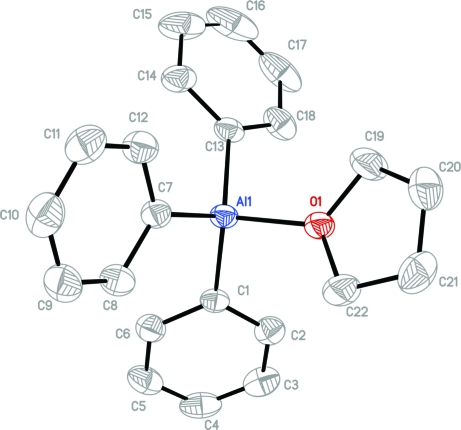
The molecular structure of the title compound with atom-numbering and displacement ellipsoids drawn at the 20% probability level.

## supplementary crystallographic information

### Comment

Triphenylaluminium was first reported 40 years ago and the solid-state study revealed a dimeric Al_2_Ph_6_ structure bridging through two phenyl groups (Malone & McDonald, 1967). For synthesis of monomeric triarylaluminium complexes, two synthetic strategies were used. The first route employed a reaction of dimesitylmercury with Al/HgCl_2_, furnishing three-coordinate trimesitylaluminium (Jerius *et al.*, 1986) which possesses a trigonal planar structure. The second synthetic route used a strategy of providing an additional neutral ligand, such as tetrahydrofuran (THF) or diethyl ether (OEt_2_), giving four-coordinate monomeric AlAr_3_(*L*) complexes (*L* = THF or OEt_2_) (Barber *et al.*, 1982; De Mel & Oliver, 1989). In addition to structural studies, organoaluminium reagents had been demonstrated as excellent nucleophiles in organic synthesis, owing to their higher reactivity and the Lewis acidity of the aluminium center. Recently, we reported applications of AlAr_3_(THF) in asymmetric aryl additions to aldehydes (Wu & Gau, 2006) and to ketones (Chen *et al.*, 2007) and in coupling reactions (Ku *et al.*, 2007). Due to their diversified applications in catalysis, we report herein the synthesis and structure of a four-coordinate triphenylaluminium compound, [Al(C_6_H_5_)_3_(OC_4_H_8_)].

The molecule of the title compound contains unsubstituted phenyl ligands and has a distorted tetrahedral geometry around the aluminium metal center (Fig. 1). The Al—O(THF) bond length of 1.8972 (13) Å is shorter by 0.08 Å than the Al—C bond distances of 1.9783 (18), 1.9800 (18) and 1.9809 (19) Å. This complex has similar Al—C bond distances with the four-coordinate (*o*-tol)_3_Al(OEt_2_) complex (Barber *et al.*, 1982). The C—Al—O bond angles in the title complex [103.39 (7), 103.90 (6) and 103.75 (7)°] are much smaller than the C—Al—C bond angles [113.25 (7), 114.23 (7) and 116.27 (8)°]. In contrast, the bulky mesityl ligands in trimesityl(tetrahydrofuran)aluminium complex (De Mel & Oliver, 1989) repel each other, giving longer Al—C bond lengths of 2.011 (7), 2.020 (7) and 2.021 (6) Å. Similarly, the Al—O(THF) bond distance of 1.969 (5) Å in the above complex is longer by 0.07 Å than the Al—O(THF) bond length in the title compound.

### Experimental

A solution of phenylmagnesium bromide (90.0 mmol) in THF (50 ml) was slowly added to a solution of AlCl_3_ (4.00 g, 30.0 mmol) in THF (20 ml) at 273 K. The mixture was stirred at room temperature for 12 h and the solvent was removed under reduced pressure to afford a residue which was extracted with toluene (2 × 40 ml). The extracts were combined and concentrated to about 50 ml. Colourless crystals of the title compound (8.92 g, 90.0% yield) were obtained by cooling the concentrated solution at 273 K. The above synthetic procedures were conducted strictly under nitrogen atmosphere. ^1^H NMR (CDCl_3_, 400 MHz): δ 7.80–7.76 (m, 6H), 7.34–7.30 (m, 9H), 4.16 (m, 4H), 2.01 (m, 4H) p.p.m. ^13^C{^1^H} NMR (CDCl_3_, 100 MHz): δ 146.74, 137.99, 127.50, 127.06, 75.59, 24.97 p.p.m. Analysis calculated for C_22_H_23_OAl: C 79.97, H 7.02%;found: C 79.44, H 6.75%.

### Refinement

All H atoms were fixed geometrically [C—H = 0.93 Å or 0.97 Å] and treated as riding, with *U*_iso_(H) = 1.2U_eq_(C). The C atoms of the tetrahydrofuran ring display large displacement parameters, but no suitable refinement model for disorder was found.

### Crystal data

**Table:**

[Al(C_6_H_5_)_3_(C_4_H_8_O)]	*F*(000) = 704
*M**_r_* = 330.38	*D*_x_ = 1.128 Mg m^−^^3^
Monoclinic, *P*2_1_/*c*	Mo *K*α radiation, λ = 0.71073 Å
Hall symbol: -P 2ybc	Cell parameters from 3804 reflections
*a* = 9.649 (2) Å	θ = 2.1–26.0°
*b* = 12.966 (3) Å	µ = 0.11 mm^−^^1^
*c* = 16.038 (4) Å	*T* = 293 K
β = 104.210 (4)°	Block, colourless
*V* = 1945.1 (8) Å^3^	0.58 × 0.42 × 0.21 mm
*Z* = 4	

### Data collection

**Table:**

Bruker SMART 1000 CCD diffractometer	3804 independent reflections
Radiation source: fine-focus sealed tube	2971 reflections with *I* > 2σ(*I*)
graphite	*R*_int_ = 0.023
φ and ω scans	θ_max_ = 26.0°, θ_min_ = 2.1°
Absorption correction: multi-scan (SADABS; Sheldrick, 1996)	*h* = −8→11
*T*_min_ = 0.799, *T*_max_ = 1.000	*k* = −15→15
10682 measured reflections	*l* = −19→17

### Refinement

**Table:**

Refinement on *F*^2^	Primary atom site location: structure-invariant direct methods
Least-squares matrix: full	Secondary atom site location: difference Fourier map
*R*[*F*^2^ > 2σ(*F*^2^)] = 0.048	Hydrogen site location: inferred from neighbouring sites
*wR*(*F*^2^) = 0.162	H-atom parameters constrained
*S* = 1.33	*w* = 1/[σ^2^(*F*_o_^2^) + (0.0861*P*)^2^] where *P* = (*F*_o_^2^ + 2*F*_c_^2^)/3
3804 reflections	(Δ/σ)_max_ = 0.001
217 parameters	Δρ_max_ = 0.27 e Å^−^^3^
0 restraints	Δρ_min_ = −0.23 e Å^−^^3^

### Special details

**Table:**

Geometry. All e.s.d.'s (except the e.s.d. in the dihedral angle between two l.s. planes) are estimated using the full covariance matrix. The cell e.s.d.'s are taken into account individually in the estimation of e.s.d.'s in distances, angles and torsion angles; correlations between e.s.d.'s in cell parameters are only used when they are defined by crystal symmetry. An approximate (isotropic) treatment of cell e.s.d.'s is used for estimating e.s.d.'s involving l.s. planes.
Refinement. Refinement of *F*^2^ against ALL reflections. The weighted *R*-factor *wR* and goodness of fit *S* are based on *F*^2^, conventional *R*-factors *R* are based on *F*, with *F* set to zero for negative *F*^2^. The threshold expression of *F*^2^ > σ(*F*^2^) is used only for calculating *R*-factors(gt) *etc*. and is not relevant to the choice of reflections for refinement. *R*-factors based on *F*^2^ are statistically about twice as large as those based on *F*, and *R*- factors based on ALL data will be even larger.

### Fractional atomic coordinates and isotropic or equivalent isotropic displacement parameters (Å^2^)

**Table:**

	*x*	*y*	*z*	*U*_iso_*/*U*_eq_	
Al1	0.39089 (5)	0.69171 (4)	0.18576 (3)	0.0508 (2)	
O1	0.38837 (12)	0.75437 (10)	0.29228 (7)	0.0623 (4)	
C1	0.27597 (17)	0.56515 (13)	0.18473 (11)	0.0540 (4)	
C2	0.2690 (2)	0.51194 (15)	0.25899 (13)	0.0668 (5)	
H2	0.3158	0.5394	0.3120	0.080*	
C3	0.1956 (3)	0.42013 (17)	0.25735 (17)	0.0818 (6)	
H3	0.1936	0.3872	0.3086	0.098*	
C4	0.1265 (2)	0.37804 (17)	0.1810 (2)	0.0880 (7)	
H4	0.0771	0.3162	0.1797	0.106*	
C5	0.1296 (2)	0.42674 (18)	0.10597 (17)	0.0836 (7)	
H5	0.0820	0.3982	0.0536	0.100*	
C6	0.2037 (2)	0.51857 (15)	0.10780 (13)	0.0672 (5)	
H6	0.2053	0.5503	0.0560	0.081*	
C7	0.30029 (18)	0.79599 (14)	0.09920 (11)	0.0573 (4)	
C8	0.1554 (2)	0.78994 (17)	0.05675 (12)	0.0699 (5)	
H8	0.1016	0.7356	0.0700	0.084*	
C9	0.0891 (2)	0.8605 (2)	−0.00356 (15)	0.0864 (7)	
H9	−0.0075	0.8535	−0.0302	0.104*	
C10	0.1641 (3)	0.9404 (2)	−0.02443 (16)	0.0907 (7)	
H10	0.1195	0.9879	−0.0658	0.109*	
C11	0.3064 (3)	0.95119 (19)	0.01558 (16)	0.0891 (7)	
H11	0.3581	1.0065	0.0019	0.107*	
C12	0.3729 (2)	0.87933 (16)	0.07661 (13)	0.0728 (5)	
H12	0.4694	0.8875	0.1032	0.087*	
C13	0.59598 (17)	0.66352 (14)	0.19566 (12)	0.0585 (4)	
C14	0.6632 (2)	0.68758 (18)	0.13121 (16)	0.0793 (6)	
H14	0.6130	0.7237	0.0831	0.095*	
C15	0.8054 (3)	0.6587 (3)	0.1368 (3)	0.1220 (12)	
H15	0.8478	0.6752	0.0924	0.146*	
C16	0.8810 (3)	0.6072 (3)	0.2061 (4)	0.1415 (18)	
H16	0.9759	0.5898	0.2100	0.170*	
C17	0.8176 (3)	0.5803 (2)	0.2712 (3)	0.1196 (13)	
H17	0.8686	0.5430	0.3183	0.144*	
C18	0.6776 (2)	0.60906 (17)	0.26599 (15)	0.0801 (6)	
H18	0.6364	0.5916	0.3108	0.096*	
C19	0.5018 (3)	0.8175 (3)	0.3436 (2)	0.1160 (11)	
H19A	0.5475	0.8572	0.3066	0.139*	
H19B	0.5734	0.7742	0.3805	0.139*	
C20	0.4384 (3)	0.8856 (2)	0.39505 (16)	0.0958 (8)	
H20A	0.4383	0.9560	0.3745	0.115*	
H20B	0.4921	0.8834	0.4547	0.115*	
C21	0.2920 (3)	0.8503 (2)	0.38737 (17)	0.1015 (8)	
H21A	0.2860	0.8115	0.4381	0.122*	
H21B	0.2275	0.9087	0.3815	0.122*	
C22	0.2540 (3)	0.7853 (2)	0.31139 (17)	0.0937 (8)	
H22A	0.1952	0.8232	0.2634	0.112*	
H22B	0.2013	0.7253	0.3223	0.112*	

### Atomic displacement parameters (Å^2^)

**Table:**

	*U*^11^	*U*^22^	*U*^33^	*U*^12^	*U*^13^	*U*^23^
Al1	0.0376 (3)	0.0578 (3)	0.0577 (3)	−0.00033 (19)	0.0129 (2)	−0.0031 (2)
O1	0.0481 (7)	0.0751 (8)	0.0667 (8)	−0.0054 (6)	0.0197 (5)	−0.0148 (6)
C1	0.0381 (8)	0.0582 (10)	0.0664 (10)	0.0042 (7)	0.0140 (7)	−0.0002 (7)
C2	0.0603 (11)	0.0681 (11)	0.0727 (12)	0.0086 (9)	0.0174 (9)	0.0077 (9)
C3	0.0814 (15)	0.0639 (12)	0.1086 (17)	0.0102 (11)	0.0395 (13)	0.0202 (12)
C4	0.0677 (13)	0.0540 (11)	0.152 (2)	−0.0030 (10)	0.0462 (15)	−0.0004 (14)
C5	0.0645 (13)	0.0739 (14)	0.1074 (17)	−0.0091 (10)	0.0115 (12)	−0.0236 (12)
C6	0.0617 (11)	0.0696 (12)	0.0696 (11)	−0.0052 (9)	0.0147 (8)	−0.0044 (9)
C7	0.0494 (10)	0.0637 (10)	0.0604 (10)	0.0024 (8)	0.0166 (7)	−0.0036 (7)
C8	0.0520 (11)	0.0782 (13)	0.0780 (13)	0.0087 (9)	0.0132 (9)	0.0022 (10)
C9	0.0616 (12)	0.1058 (18)	0.0869 (14)	0.0202 (13)	0.0090 (10)	0.0061 (13)
C10	0.0918 (17)	0.0995 (17)	0.0817 (14)	0.0332 (14)	0.0230 (12)	0.0228 (12)
C11	0.0990 (19)	0.0791 (14)	0.0950 (16)	0.0011 (13)	0.0351 (14)	0.0210 (12)
C12	0.0638 (12)	0.0744 (12)	0.0788 (12)	−0.0017 (10)	0.0149 (9)	0.0058 (10)
C13	0.0416 (8)	0.0606 (10)	0.0736 (11)	−0.0031 (8)	0.0149 (7)	−0.0177 (8)
C14	0.0639 (12)	0.0857 (14)	0.0985 (15)	−0.0117 (10)	0.0397 (11)	−0.0284 (12)
C15	0.0816 (19)	0.121 (2)	0.192 (3)	−0.0240 (18)	0.088 (2)	−0.069 (2)
C16	0.0459 (14)	0.126 (3)	0.248 (5)	0.0064 (16)	0.027 (2)	−0.093 (3)
C17	0.0632 (16)	0.0994 (19)	0.167 (3)	0.0286 (14)	−0.0274 (18)	−0.054 (2)
C18	0.0604 (12)	0.0780 (14)	0.0911 (14)	0.0149 (10)	−0.0020 (10)	−0.0159 (11)
C19	0.0805 (16)	0.150 (3)	0.124 (2)	−0.0434 (17)	0.0365 (15)	−0.0780 (19)
C20	0.115 (2)	0.0881 (16)	0.0816 (15)	−0.0032 (15)	0.0194 (13)	−0.0192 (12)
C21	0.125 (2)	0.0909 (17)	0.1067 (19)	0.0074 (16)	0.0622 (16)	−0.0156 (14)
C22	0.0641 (13)	0.122 (2)	0.1058 (17)	−0.0033 (13)	0.0421 (12)	−0.0335 (15)

### Geometric parameters (Å, °)

**Table:**

Al1—O1	1.8972 (13)	C11—C12	1.389 (3)
Al1—C1	1.9783 (18)	C11—H11	0.93
Al1—C13	1.9800 (18)	C12—H12	0.93
Al1—C7	1.9809 (19)	C13—C14	1.384 (3)
O1—C19	1.450 (2)	C13—C18	1.398 (3)
O1—C22	1.460 (2)	C14—C15	1.404 (4)
C1—C2	1.392 (3)	C14—H14	0.93
C1—C6	1.397 (3)	C15—C16	1.346 (5)
C2—C3	1.382 (3)	C15—H15	0.93
C2—H2	0.93	C16—C17	1.378 (5)
C3—C4	1.357 (3)	C16—H16	0.93
C3—H3	0.93	C17—C18	1.384 (4)
C4—C5	1.365 (3)	C17—H17	0.93
C4—H4	0.93	C18—H18	0.93
C5—C6	1.385 (3)	C19—C20	1.443 (3)
C5—H5	0.93	C19—H19A	0.97
C6—H6	0.93	C19—H19B	0.97
C7—C12	1.384 (3)	C20—C21	1.461 (4)
C7—C8	1.399 (2)	C20—H20A	0.97
C8—C9	1.371 (3)	C20—H20B	0.97
C8—H8	0.93	C21—C22	1.452 (3)
C9—C10	1.351 (4)	C21—H21A	0.97
C9—H9	0.93	C21—H21B	0.97
C10—C11	1.372 (4)	C22—H22A	0.97
C10—H10	0.93	C22—H22B	0.97
			
O1—Al1—C1	103.39 (7)	C11—C12—H12	118.9
O1—Al1—C13	103.90 (6)	C14—C13—C18	116.2 (2)
C1—Al1—C13	113.25 (7)	C14—C13—Al1	122.74 (16)
O1—Al1—C7	103.75 (7)	C18—C13—Al1	120.78 (16)
C1—Al1—C7	114.23 (7)	C15—C14—C13	121.4 (3)
C13—Al1—C7	116.27 (8)	C15—C14—H14	119.3
C19—O1—C22	108.11 (17)	C13—C14—H14	119.3
C19—O1—Al1	125.33 (13)	C14—C15—C16	120.6 (3)
C22—O1—Al1	121.01 (12)	C14—C15—H15	119.7
C2—C1—C6	115.02 (17)	C16—C15—H15	119.7
C2—C1—Al1	123.27 (13)	C15—C16—C17	119.9 (3)
C6—C1—Al1	121.56 (14)	C15—C16—H16	120.0
C3—C2—C1	122.9 (2)	C17—C16—H16	120.0
C3—C2—H2	118.6	C18—C17—C16	119.6 (3)
C1—C2—H2	118.6	C18—C17—H17	120.2
C4—C3—C2	120.0 (2)	C16—C17—H17	120.2
C4—C3—H3	120.0	C17—C18—C13	122.2 (3)
C2—C3—H3	120.0	C17—C18—H18	118.9
C3—C4—C5	119.8 (2)	C13—C18—H18	118.9
C3—C4—H4	120.1	O1—C19—C20	107.6 (2)
C5—C4—H4	120.1	O1—C19—H19A	110.2
C6—C5—C4	120.1 (2)	C20—C19—H19A	110.2
C6—C5—H5	119.9	O1—C19—H19B	110.2
C4—C5—H5	119.9	C20—C19—H19B	110.2
C5—C6—C1	122.2 (2)	H19A—C19—H19B	108.5
C5—C6—H6	118.9	C19—C20—C21	107.3 (2)
C1—C6—H6	118.9	C19—C20—H20A	110.3
C12—C7—C8	115.23 (18)	C21—C20—H20A	110.3
C12—C7—Al1	123.50 (14)	C19—C20—H20B	110.3
C8—C7—Al1	121.27 (15)	C21—C20—H20B	110.3
C9—C8—C7	122.9 (2)	H20A—C20—H20B	108.5
C9—C8—H8	118.6	C22—C21—C20	107.0 (2)
C7—C8—H8	118.6	C22—C21—H21A	110.3
C10—C9—C8	120.1 (2)	C20—C21—H21A	110.3
C10—C9—H9	120.0	C22—C21—H21B	110.3
C8—C9—H9	120.0	C20—C21—H21B	110.3
C9—C10—C11	119.8 (2)	H21A—C21—H21B	108.6
C9—C10—H10	120.1	C21—C22—O1	106.4 (2)
C11—C10—H10	120.1	C21—C22—H22A	110.5
C12—C11—C10	119.8 (2)	O1—C22—H22A	110.5
C12—C11—H11	120.1	C21—C22—H22B	110.5
C10—C11—H11	120.1	O1—C22—H22B	110.5
C7—C12—C11	122.2 (2)	H22A—C22—H22B	108.6
C7—C12—H12	118.9		

### Hydrogen-bond geometry (Å, °)

**Table:**

*D*—H···*A*	*D*—H	H···*A*	*D*···*A*	*D*—H···*A*
C16—H16···Cg1^i^	0.93	2.78	3.654 (4)	156
C19—H19A···Cg1^ii^	0.97	2.81	3.600 (4)	139

Symmetry codes: (i) *x*+1, *y*, *z*; (ii) −*x*+1, *y*+1/2, −*z*+1/2.
